# Case report: Identification and clinical phenotypic analysis of novel mutation of the *PPP1CB* gene in NSLH2 syndrome

**DOI:** 10.3389/fnbeh.2022.987259

**Published:** 2022-09-08

**Authors:** Xuemei He, Xiuli Ma, Jing Wang, Zhuo Zou, Haoyu Huang, Jian Ren, Chunming Liu, Nan Zheng, Jing Ma, Yun Liu

**Affiliations:** ^1^Department of Rehabilitation, Kunming Children's Hospital, Kunming Medical University, Yunnan, China; ^2^Department of Otolaryngology, Head and Neck Surgery, Kunming Children's Hospital, Kunming Medical University, Yunnan, China

**Keywords:** *PPP1CB*, Noonan syndrome with loose anagen hair-2, developmental delay, next-novel mutation of the *PPP1CB* gene 2 generation sequencing, DNA mutation analysis

## Abstract

**Objective:**

To screen and analyze the genetic mutations in the *PPP1CB* gene in a patient with Noonan syndrome with loose anagen hair-2 (NSLH2) in Yunnan Province, China and explore the possible molecular pathogenesis.

**Methods:**

After obtaining informed consent, we collected the patient's medical history and carried out physical and laboratory examinations for the NSLH2 proband and the family members. Genomic DNA was extracted from the peripheral blood of all individuals. The coding regions including all pathogenic exons, parts of introns, and promoters of genes were sequenced by next-generation sequencing. Pathogenic mutations, which were detected in the probands and their parents, were verified by Sanger sequencing.

**Results:**

The clinical manifestations of NSLH2 included prominent forehead, yellowish hair, slightly wide eye distance, sparse eyebrows, bilateral auricle deformity, reduced muscle tension, and cardiac and visual abnormalities. The proband carried a c.371A>G mutation in exon 3 of *PPP1CB*, which is a missense mutation. This was a *de novo* mutation as the parents of the proband showed no mutation at this site.

**Conclusion:**

In this study, we identified a novel mutation of *PPP1CB*, which enriched the mutation spectrum of the *PPP1CB* gene and provided a basis for the diagnosis of NSLH2.

## Introduction

Noonan syndrome-like disorder with loose anagen hair-2 (NSLH2, OMIM: 617506) is an autosomal dominant inherited disorder. It is a class of RAS signaling pathway-related syndromes (RASopathies) resulting from genetic variations in the RAS/MAPK pathways (Mazzanti et al., 2003). The presently reported pathogenic genes are *SHOC2* and *PPP1CB*. The *SHOC2* gene mutation causes type 1 Noonan syndrome with loose anagen hair (NSLH1) and the *PPP1CB* gene mutation causes NSLH2. Protein phosphatase 1 (PPP1), a major type 1 serine/threonine phosphatase, is widely expressed and regulates a variety of cellular functions, including metabolism, cell division, and muscle contraction (Barker et al., 1994). In addition to certain characteristics of Noonan syndrome, NSLH2 is characterized by chronic hair loss due to easily pulled out and thinning hair, slow growth, and pale color. Most NSLH2 patients are short in stature owing to lack of growth hormone (Cordeddu et al., 2009). This disease can also involve the skin and nervous, cardiovascular, and skeletal systems.

In this study, we identified and analyzed the genetic mutations of a pediatric patient with a clinical diagnosis of “general developmental delay, visual dysplasia, ventricular septal defect, nodal cause to be investigated” and his parents by using total exome sequencing technology. The sequencing results showed that the child carried the c.371A>G (exon 3) mutation in *PPP1CB*. This study enriched the mutation spectrum of the *PPP1CB* gene and provided a certain reference for the diagnosis of NSLH.

## Case presentation

### Case

The proband was a female patient aged 6 months and 14 days with general developmental delay, visual dysplasia, ventricular septal defect, and nodding of unknown cause who was admitted to our hospital in Yunnan Province in 2019. Clinical data of the patient and her families were collected, and comprehensive physical examination and intelligence assessment were carried out. Computed tomography (CT) of the temporal bone and magnetic resonance imaging (MRI) of the skull were performed. This study was approved by the Medical Ethics Committee of Kunming Children's Hospital. The genetic diagnosis was approved by the child's family members, and informed consent was signed.

### Genetic testing methods

Peripheral blood of the patient and her family members was collected to construct a DNA extraction. Tissue genomic DNA was extracted using the Blood Genome Column Medium Volume Extraction Kit (Convoy Century), following the kit instructions. All exon sequences related to clinical diseases were captured and hybridized to enrich the target region sequences. Double-end sequencing with the aid of low depth whole genome sequencing based on the Illumina technology sequencing platform. The filtered sequences were aligned to the Human Genome Reference Sequence (UCSC, HG19) of the NCBI database using BWA software (http://bio-bwa.sourceforge.net/), and any redundant data in the PCR process were removed. The relevant information of single nucleotide polymorphism (SNP) and insertion-deletion mutation (Indels) was analyzed using GATK software. All SNPs and Indels were annotated by Annovar software. Mutant loci with a frequency of <0.05 were screened out from the standard databases including the 1000 Genome Project, Exome Variant Server, and Exec. SIFT. Polyphen-2 Mutation Taster and GERP++ software were used to predict the pathogenicity and conservatism of missense mutations. SPidex software was used to analyze the pathogenicity of shear site changes. Sanger sequencing verified the second-generation sequencing mutation sites. Mutation pathogenicity was analyzed according to the ACMG guidelines. The relation of genotype to phenotype was conducted.

### Clinical data analysis

The patient was a girl aged 6 months and 14 days who was admitted to our hospital on July 14, 2020, for “developmental delay.” The baby was delivered by cesarean section owing to uterine scarring at 38 weeks^+6^ of gestation, with a birth weight of 3,100 grams. Her mother denied any history of abnormal pregnancy or perinatal asphyxia and rescue; moreover, the patient's jaundice was not severe in the neonatal period. The child's psychomotor development was delayed since childhood, and the stability of the head in the vertical plane was poor when examined by the doctor. She could only turn over from the supine position to the lateral position, and her hands could not take objects actively. Her eyes followed the red ball <90°, and she was not good at following the sources of sound. She often sucked her fingers. Her family members complained that her facial expression was slightly tense when she changed from the lying position to the sitting position without the back chair, and occasionally exhibited double oblique vision. She was in good spirits and sleeping well, but she had poor appetite and indigestion often, milk flap in stool, and would often vomit milk before the age of 6 months. After 6 months, her symptoms began to ease. Family members reported that she exhibited frequent nodding behavior, but no symptoms of epileptic seizure such as mouth and face blue, hug shape, limb shaking, eyes staring, foaming etc.

The patient's elder sister was 3.5 years old, and her development was consistent with age. Her parents were in good health and were not closely related. There was no similar medical history in her family.

### Physical examination analysis

**Figure 1:**
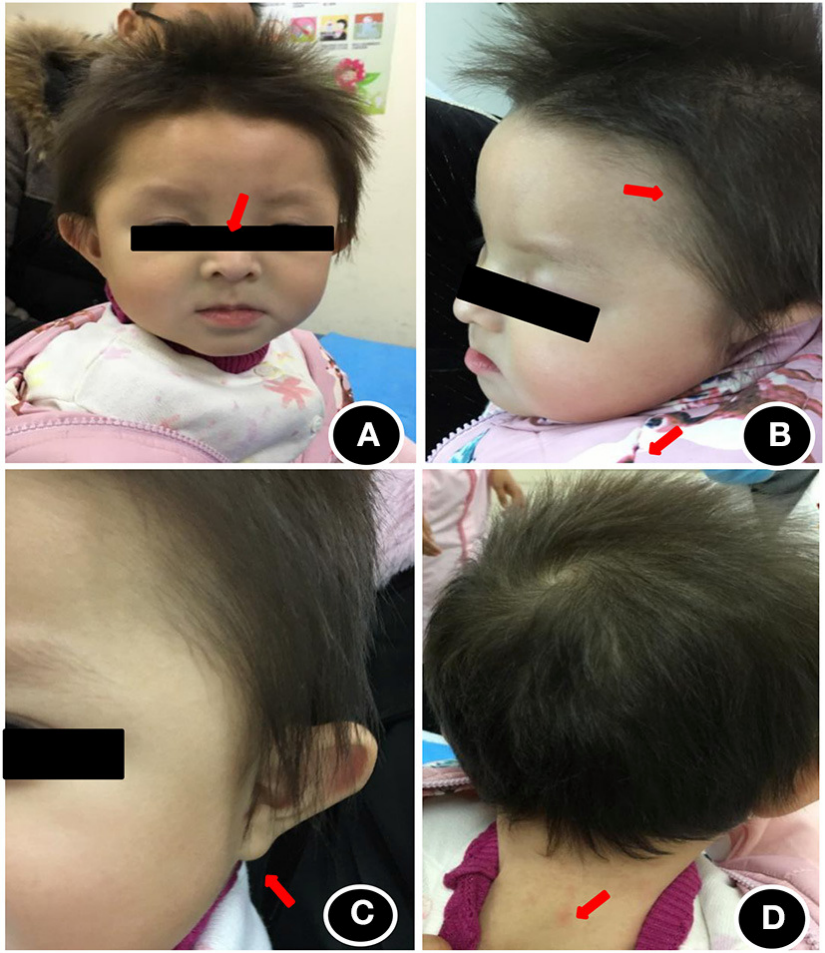
Head and facial features of the child at the age of 1 year. **(A)** Wide eye spacing, sparse eyebrows, light color; **(B)** Loose hair, sparse hair, light color; **(C)** abnormal auricle appearance; **(D)** dry and eczema-prone skin.

The patient showed stable vital signs and the head circumference was 41 cm. The anterior fontanelle was flat and soft with a size of about 0.5 × 0.5 cm. As shown in Figure 1, she exhibited some unique head and facial features, including prominent forehead, white skin, yellowish hair color, slightly wider eye distance, scant eyebrows, light complexion, bilateral auricle shape abnormity, Both pupils are equal in size and round, and dry skin that was prone to eczema (Figure 1). There was no desquamation, no light reflex, no congestion in the pharynx, no resistance in the neck, and no obvious abnormality in the heart, lung, and abdomen. Extremities did not exhibit the symptoms of edema and cyanosis. The muscle volume of the extremities was normal, but the muscle tone was reduced. The knee reflex was present. Her hands are less flexible for active picking and she has less movement in the midline position of her body. Prone position could be assumed with elbow support, but the patient could lean forward to sit independently only for some time. Standing double lower limbs will support, but she could not jump.

### Auxiliary examination analysis

**Figure 2:**
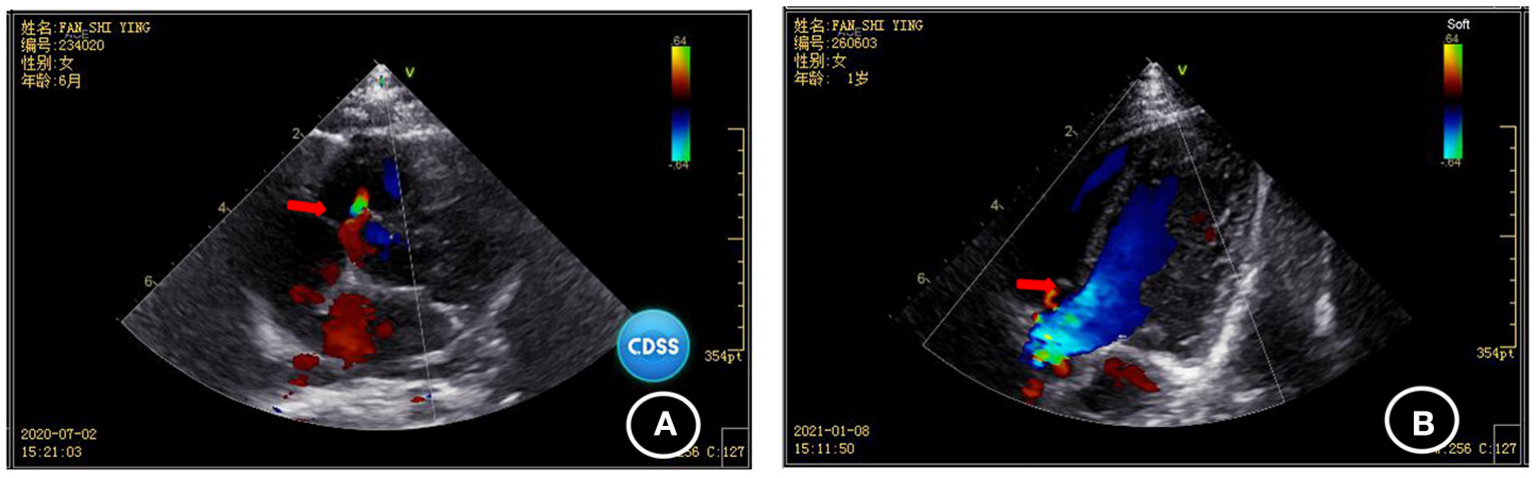
Comparison of the results of two cardiac ultrasonographies of the child **(A)** ventricular septal defect and pseudomonal tumor formation (rupture of about 2.5 mm and 1.8 mm) were found in July 2020; **(B)** Ventricular septal defect and pseudo membrane tumor formation (1.3 mm rupture) were found by heart color doppler ultrasound in January 2021.

**Figure 3:**
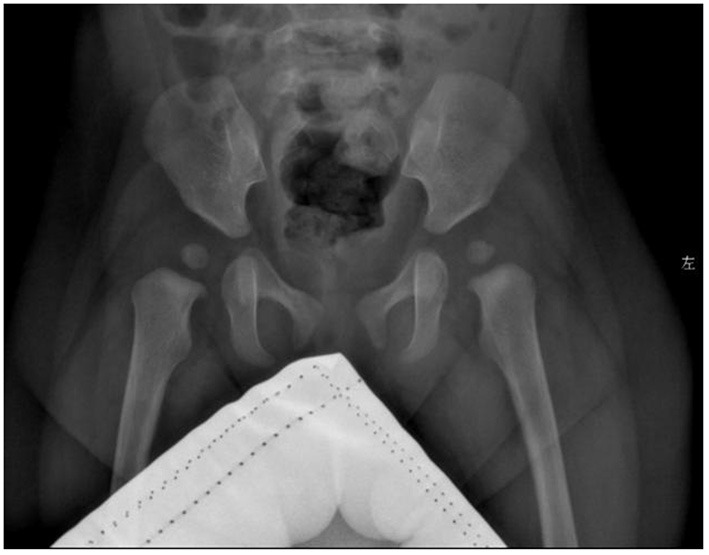
Radiographs of the child's hip joint show slightly flat and shallow bilateral acetabular fossa and enlarged bilateral acetabular angle.

Blood biochemical indices, thyroid function, and vitamin D level were normal in July 2020. The electroencephalogram was also normal. Chromosomal karyotyping showed 46, XX. Ultrasonography of the heart revealed ventricular septal defect and pseudomonal tumor formation (rupture of about 2.5 mm and 1.8 mm) (Figure 2A). Radiography of the hip joint showed bilateral hip dysplasia (Figure 3). Brain MRI showed slightly wider extracerebral space in the bilateral frontotemporal region; no definite abnormality was found in the rest of the brain. The results of auditory brain stem response were Transient Evoked Otoacoustic Emission (TEOAE) in both ears. The auditory brain stem response was as follows: The click waveform of 70 dBnHL in both ears was well-differentiated, and the interval and latency were normal. The response threshold was 20 dBnHL in the right ear and 25 dBnHL in the left ear.

### Griffiths developmental assessment

Motor:score 3.8 points, developmental age 3 month, developmental level percentile1–2.5%; Personal-society: score 3.4 points, developmental age 2–2.5m, developmental level percentile <1%; Language: score 2.1 points, developmental age 1 m, developmental level percentile <1%. Hand-eye coordination:score 2.4 points, developmental age 1.5–2m, developmental level percentile <1%; Performance:score 2.7 points, developmental age 2.5m, developmental level percentile <1%. These results indicate that the development of each energy region was delay. Fundus examination revealed retinal exudation around the right eye and no obvious abnormality in the left eye. Fundus examination in August 2020 showed no abnormality in either eye.

Follow-up: (i) The follow-up to October 1, 2020, 9 months of age 27 days, children from 7.8 kg weight increased to 8.5 kg, head circumference has no obvious change, will each hand holding a toy, can sit alone 10 s, the light can be caught and beat 180°, rattle loss will be looking for, the reaction is a bit slow, call names I will turn, slower reaction, will imitate poop-poop sound, cookies from hello, like to play with a rattle, fine motor, chase, chase before listening, language is improved. (ii) In the follow-up to December 24, 2020, the child could sit independently for 2–3 min, pick things with both hands, and laugh when communicating with others. Gross motor, fine motor, cognition, language, and social interaction were better than before. (iii) Follow-up was carried out until January 8, 2021. At that time, the child was 1 year and 9 days old. The child could sit independently for 7–8 min and could turn over, but not crawl; Will take the initiative, will hold their toys, model bell ringing, will tear paper, not the thumb and forefinger pinch pills; Listen to the sound will look for the sound source, will change hands; Made will laugh aloud, will unconsciously shout “mom”, will lift surface play peek-a-boo, can recognize a person, sometimes nod, spirit, poor diet, prone to indigestion, defecate have milk disc, stool stem node, to sleep, sometimes nod, no opening week and was blue, no hug, no limb jitter, unique eye gaze, no saliva and so on. Gross motor, fine motor, cognition, language, and social skills showed progress compared to before. The follow-up to January 2021, her EEG was normal.

Cardiac ultrasonography in January 2021 revealed ventricular septal defect and pseudo membrane neoplasm (1.3 mm rupture) (Figure 2B). The GDF showed the following results:

Exercise: total bare 7, developmental age 6.5–7 m, percentile 1–2.5%; Personal-society: total bare value 6.5, developmental age 5–5.5 m, percentile <1%; Language: total bare 6.5, developmental age 6–6.5 m, percentile 1–2.5%; Hand-eye coordination: total bare 6.5, developmental age 6.5–7 m, percentile 1–2.5%; and Performance: Total bare 6.5, developmental age 6.5–7 m, percentile 1–2.5%. The results of her development evaluation indicate that the development of each area is deficient, but the overall development level was slightly improved. Evaluation of bone age indicated that her bone age was equivalent to 2 years. Radiographs of the hip joint showed that the bilateral acetabular fossa was slightly flat and shallower, and the bilateral acetabular angle was enlarged.

### Genetic test results

**Figure 4:**
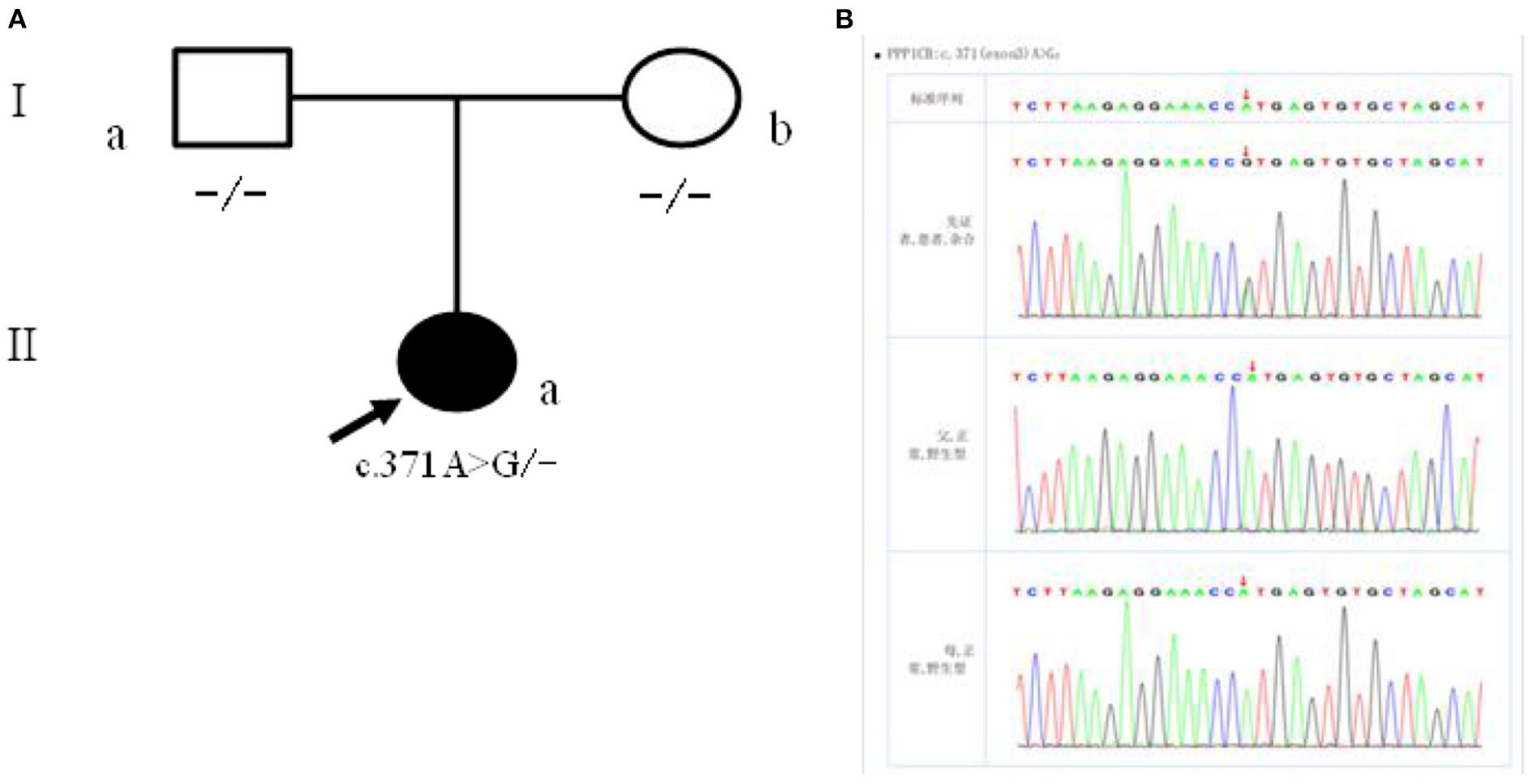
**(A)** Children with family figure for NSLH2 clinical phenotype. Her father (I-a) and mother (I-b) had normal clinical manifestations; **(B)** children with children and parents Sanger sequencing diagram (II-a) for c. 371 A>G heterozygous mutations. Her father (I-a) and mother (I-b) the site are for the wild type.

The patient had a novel heterozygous mutation in exon 3 of the coding region of *PPP1CB* (CHR2-29001861), and the nucleotide 371 changed from A to G (C. 371A → G) (Figure 4A), resulting in the mutation of the amino acid at the 124th position from histidine to arginine (p.H124R). This is a missense mutation and predicts a change of protein function. Sanger sequencing verified that the child's parents did not carry the mutation at the site, which was a spontaneous mutation (Figure 4B), according to the ACMG guidelines. The mutation is classified as “class 2 - possibly pathogenic.”

**Figure 5:**
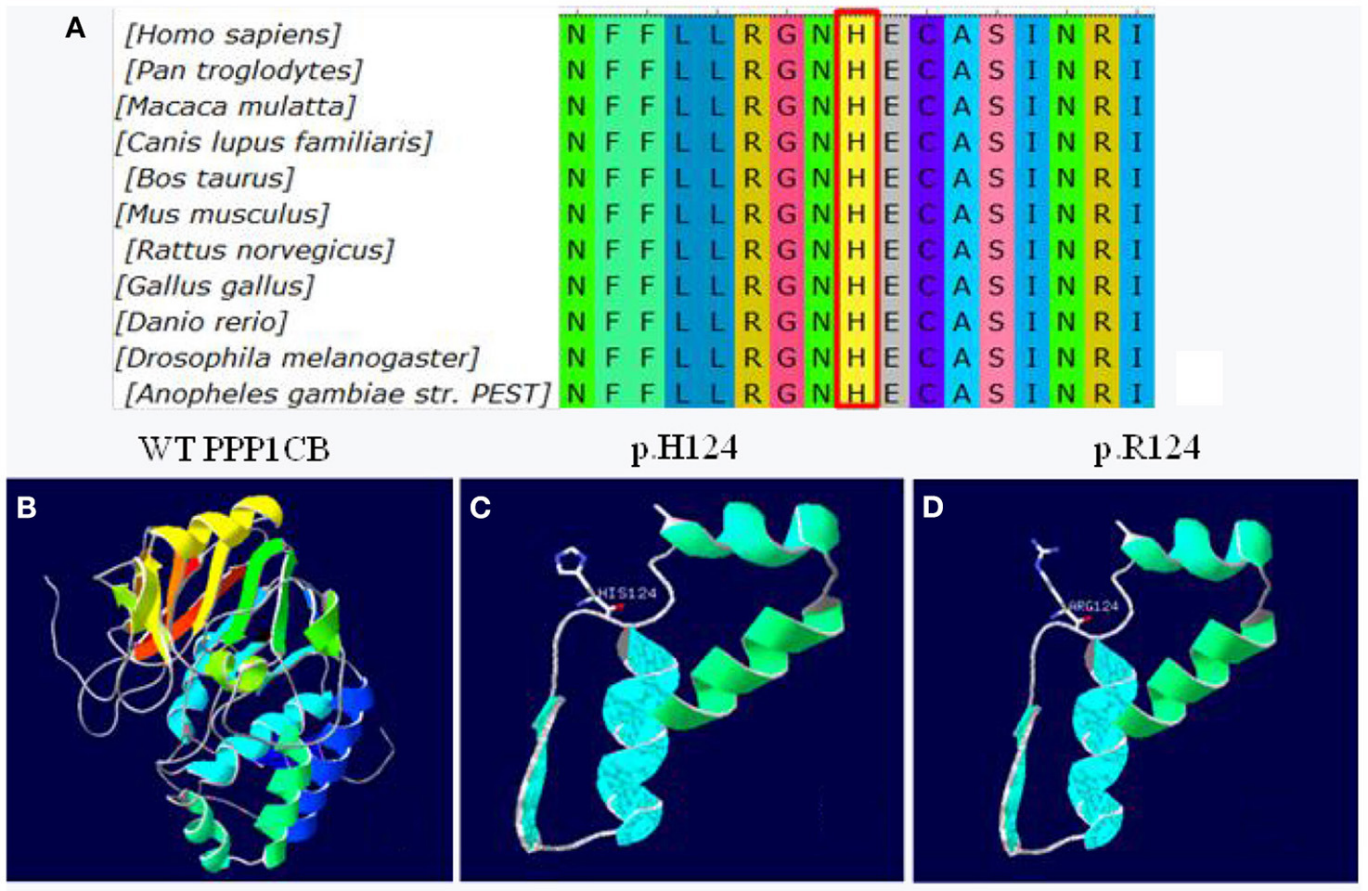
Conservation and 3D molecular model of *PPP1CB* mutations. **(A)** Protein alignment showing *PPP1CB* p.H124 occurred at evolutionarily conserved amino acids (in red box) across 11 genera; **(B)** Wild type of *PPP1CB*; **(C)** Three-dimensional molecular models of wild-type protein at site p.H124; **(D)** Abnormal structure of mutant-type protein.

The missense mutation c.371A>G was predicted to result in a histidine to arginine substitution at codon 124. The alignment of *PPP1CB* from different genera namely *Homo, Pan, Macaca, Canis, Bos, Mus, Rattus, Gallus, Danio, Drosophila*, and *Anopheles* is shown in Figure 5A. The three-dimensional structures of wild-type *PPP1CB* was simulated according to the crystal structure (Figure 5B). The missense variant (p.H124R) was predicted to perturb protein structure because of the substitution of histidine by arginine. In this respect, our prediction study revealed that the novel mutation possibly lead to protein dysfunction (Figures 5C,D).

## Discussion

Ras/mitogen-activated protein Kinase (RAS/MAPK) is one of the most important signaling pathways involved in the regulation of cell proliferation, survival, apoptosis, differentiation, immune response, and nervous system function (Zhang and Zeng, 2020). RAS signaling pathway-related syndromes are a group of syndromes caused by germline mutations in genes affecting the RAS mitogen-activated protein kinase (MAPK) pathway (Schubbert et al., 2007; Matozaki et al., 2009; Motta et al., 2020). RAS signaling pathway-related syndromes are a group of diseases caused by gene mutations in the RAS/MAPK pathway, including Noonan syndrome (NS), Noonan-like syndrome with multiple lentigines, NSML), NSLH, Capillary malformation-arteriovenous malformation (CM-AVM), Costello syndrome (CS), Cardio-facio-cutaneous syndrome (CFCS), neurofibromatosis type 1 (NF1), and Legius syndrome (LS). The overall incidence of RAS signal-associated syndromes in live births has been reported to be as high as 1 in 1250-700 (Wright and Kerr, 2010). In addition, the clinical manifestations of these diseases such as cardiovascular abnormalities, skin abnormalities, special facial features, neurological abnormalities, and varying degrees of intellectual disability overlap to a certain extent. It is difficult to diagnose these diseases only through clinical manifestations. Currently, whole exome sequencing has played a role in identifying the genetic etiology of RAS signaling pathway-related syndromes (Gripp et al., 2016).

To date, 23 genes related to RAS signaling pathway-related syndrome have been reported in literature; these are *PTPN11, SOS1, RAF1, NRAS, CBL, NF1, RASA1, HRAS, BRAF, MAP2K1, MAP2K2, KRAS, SPRED1, SHOC2, RRAS, RIT1, RASA2, SOS2, MAP3K8, SPRY1, MYST4, LZTR1*, and *A2ML1* (Schubbert et al., 2007; Matozaki et al., 2009; Wright and Kerr, 2010; Gripp et al., 2016; Umeki et al., 2018; Motta et al., 2020; Zhang and Zeng, 2020). Among them, 16 genes are related to Noonan syndrome (Li et al., 2020), and two genes, namely *SHOC2* and *PPP1CB*, are related to Noonan syndrome with birth hair loosening (Haverfield et al., 2018).

Protein phosphatase 1 (PPP1), a major type 1 serine/threonine phosphatase, is widely expressed and regulates a variety of cellular functions including metabolism, cell division, and muscle contraction (Barker et al., 1994). PPP1 is a serine/threonine specific phosphatase that balances serine/threonine kinases to regulate the activation of signaling proteins such as mitogen-activated protein kinases in the RAS/MAPK pathway. PPP1 is a holoenzyme composed of a catalytic subunit (PPP1C) and a regulatory subunit (PPP1R). PPP1 has three catalytic subunits (PPP1C), namely alpha subunit (encoded by *PPP1Ca*), beta subunit (encoded by *PPP1CB*), and gamma subunit (encoded by *PPP1Cc*), and selective splicing within each site produces multiple PPP1 holoenzymes and leads to different functions. Differential expression of the PPP1c subtype depends on cell type or tissue or even cell location, but they have similar functional properties *in vitro*. Many studies have demonstrated the multiple roles of PPP1c subtype in the regulation of cell function. PPP1CA has been widely studied for its role in the cell cycle and apoptosis of immune cells. The PPP1CB subtype is muscle specific and involved in glycogen metabolism and muscle contraction.

Recently, the role of *PPP1CB* in cardiomyocytes has also been demonstrated: it is a myosin light chain phosphatase responsible for transient Ca2+ increase and the increase of cell shortening, and *PPP1CC*'s role in the regulation of mitosis and metabolic glutamate receptor inactivation has recently received attention (Dard et al., 2018; Degirmenci et al., 2020). *SHOC2* is a widely expressed protein that is rich in leucine repeats, which interacts with the catalytic subunit of PPP1. PPP1c plays an important role in the regulation of Ras /MAPK pathway; moreover, it also forms a complex with *SHOC2*, which is stimulated by MRAS and dephosphorylates RAFs at a serine inhibition site, thereby activating the signaling cascade. Although some studies have speculated that mutations in *PPP1CB* may lead to the activation of MAPK through the activation of RAF, further studies are needed to confirm this conclusion, e.g., amino acid changes resulting from *PPP1CB* mutation may result in enhanced substrate binding of the PP1/SHOC2 complex or prolonged activation after stimulation (Young et al., 2018).

NSLH2 is also known as Mazzanti syndrome, and its main clinical features are loose hair, relative giant, growth hormone deficiency, and low intelligence (Li et al., 2020). Clinical reports are based on this syndrome's unique hair manifestations, combined with the findings of growth hormone deficiency and other typical Noonan-syndrome features. Loose growing hair is characterized by easily pluckable, sparse, thin, slow-growing, and irregularly textured hair, which is caused by abnormal hair bulbs lacking internal and external root sheaths. Most patients with NSLH2 have short stature due to growth hormone deficiency, but our patient has not yet shown this clinical symptom. Special facial features include big head; prominent forehead; wide eye spacing; drooping eyelids; flappy and low-set ears with oval helices often accompanied by thickening; short nose with a low bridge; short neck; and neck webbing. The facial features of patients of different ages are different, which are more prominent in infancy and early and middle childhood; these characteristic facial features tend to become increasingly untypical with age. The disease can also involve other ectodermal tissue such as the skin, and some patients have eczema, ichthyosis, and hair keratosis. Some other patients have congenital heart disease. Cardiovascular system involvement may be manifested as loss of the atrial or ventricular septum, mitral/tricuspid valve dysplasia, pulmonary artery stenosis, and/or cardiac hypertrophy. Some patients may also show abnormal skeletal development such as shield chest, chicken chest, funnel chest, and elbow valgus. Central nervous system involvement may be manifested as intellectual disability, often accompanied by attention deficit/hyperactivity disorder (Zarbo and Shwayder, 2018). Feeding difficulties are common in infants and young children.

**Figure 6:**
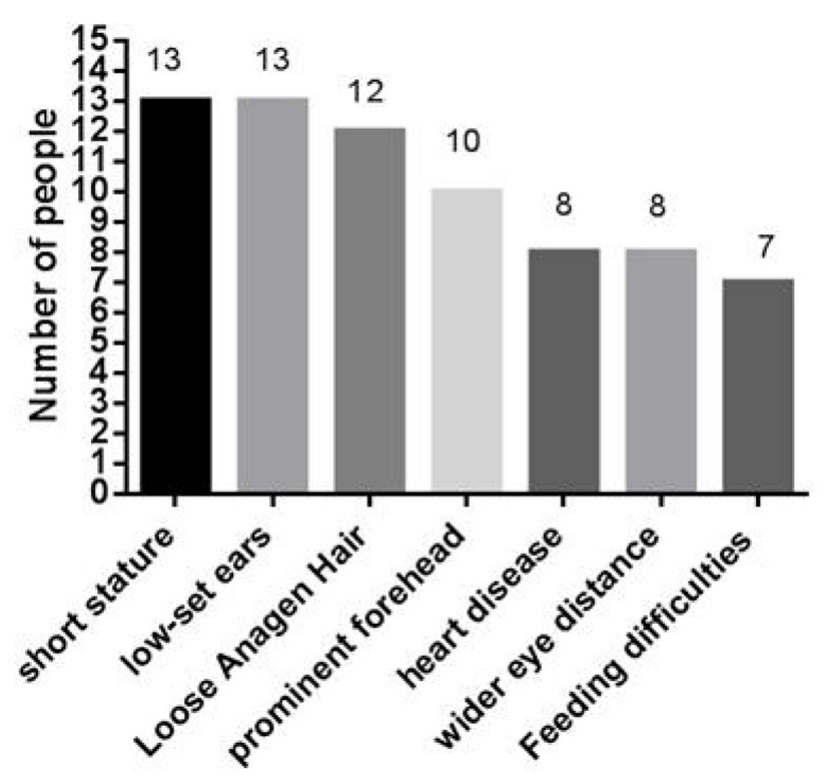
The type of NSLH2 clinical manifestations in 17 cases caused by PPP1CB gene mutation.

At present, 16 cases of *PPP1CB* gene mutation type 2 Noonan-like syndrome associated with birth hair loosening have been reported worldwide. Of these, two cases are reported in China (Zhou et al., 2020); ours is the third reported case in China. As shown in Table 1, there are seven types of gene mutations reported in 16 patients (Matozaki et al., 2009; Zambrano et al., 2017; Lin et al., 2018; Zhou et al., 2020; Maruwaka, 2022; 21). c.146C>G (p.Pro49Arg) is the hot spot of *PPP1CB* gene mutations (Zambrano et al., 2017). The mutation in this case was c.371A>G (p.His124Arg), which is a novel mutation to the best of our knowledge (Table 1). The main clinical manifestation of the 17 NSLH2 patients reported thus far (including our patient) includes unusual facial features, short stature, chest deformity, and congenital heart disease. Among them, 76.5% patients had short stature and low ear position; 70% patients had loose hair; 58% had forehead protrusion; and 47% had heart disease, wide-set eyes, and feeding difficulties (Figure 6).

**Table 1:** Basic information of the 17 cases of NSLH2 caused by *PPP1CB* gene mutation reported in literature.

**SN**	**Sex**	**Nationality**	**Base sequence**	**Amino acid**	**PMID**
1	Female	China	c.146C>G	p.Pro49Arg	PMID: 32476286
2	Male	China	c.548A>C	p.Glu183Ala	PMID: 30236064
3	Male	America	c.146G>C	p.Pro49Arg	PMID: 27264673
4	Male	America	c.166G>C	p.Ala56Pro	PMID: 27264673
5	Female	America	c.146G>C	p.Pro49Arg	PMID: 27264673
6	Female	America	c.146G>C	p.Pro49Arg	PMID: 27264673
7	Male	Brazil	c.146G>C	p.Pro49Arg	PMID: 28211982
8	Male	America	c.146C>G	p.Pro49Arg	PMID: 27868344
9	Male	America	c.146C>G	p.Pro49Arg	PMID: 27681385
10	Female	America	c.146C>G	p.Pro49Arg	PMID: 27681385
11	Male	America	c.146C>G	p.Pro49Arg	PMID: 27681385
12	Male	America	c.146C>G	p.Pro49Arg	PMID: 27681385
13	Male	America	c.548A>C	p.Glu183Ala	PMID: 27681385
14	Male	America	c.548A>T	p.Glu183Val	PMID: 27681385
15	Male	America	c.754G>T	p.Asp252Tyr	PMID: 27681385
16	Male	America	c.820G>A	p.Glu274Lys	PMID: 27681385
17	Female	America	c.371A>G	p.His124Arg	This case

The diagnosis of NSLH2 was confirmed by combining the results of genetic testing. Our patient had hip dysplasia, which was not found in previous reports. Further, most patients with NSLH2 had low ear position, but our patient did not, rather only bilateral auricle shape abnormality. Until now, no symptoms such as short stature, slow growth, and thoracic deformity have been found in the patient. Cerebellar tonsil hernia, Chiari malformation, or changes in corpus callosum have not been seen by cranial NMR. This may be because the patient is still young and has not yet shown other possible symptoms of the disease; hence, continued follow-up and preventive treatment for some of the predicted dysfunction may be required.

The diagnosis of NSLH2 is based on the patient's history, physical examination, genetic testing, and other auxiliary tests. After the definite diagnosis of NSLH2, the evaluation of cardiovascular system, endocrine system, skeletal system, digestive system, nerve, vision, skin, hearing and growth and development should be improved (Li et al., 2020). At present, there is no specific treatment for NSLH2, and symptomatic treatment is still the first choice of therapy. Cardiovascular system involvement may manifest as atrial or ventricular septal defects, mitral/tricuspid valve dysplasia, pulmonary artery stenosis, and cardiac hypertrophy. Regular follow-up, drug therapy, interventional therapy, or surgical operation should be selected according to the condition and severity (Maruwaka, 2022). Because most NSLH2 patients have problems such as overall stunting and short stature, the nutritional status and feeding conditions should be followed-up long-term, and regular nutritional assessments and timely interventions should be carried out. In 2007, the guidelines of the US Food and Drug Administration and the Pediatric Endocrinology, Genetics, and Metabolism Group of the Chinese Medical Association used recombinant human growth hormone (rhGH) for the treatment of short stature caused by Noonan syndrome (Degirmenci et al., 2020). In 2020, a study used rhGH to treat a patient with NSLH2 type 2 caused by *PPP1CB* mutation, and the results showed that the linear growth of the patient had improved (Zhou et al., 2020).

Among the 16 patients reported, one patient developed severe intractable epileptic convulsions due to a mutation in the *PPP1CB* gene C.548A>C (P.Glu183Ala). The patient's seizures were barely controlled by conventional antiepileptic drugs, but were eventually relatively controlled by a ketogenic diet. It is suggested that NSLH2 may cause RAS/MAPK-related epilepsy, and ketogenic diet may have a certain effect on *PPP1CB*-associated Noonan syndrome manifested as infantile spasm (Lin et al., 2018). The family members of the patient in this study complained that the patient had frequent nodding, and regular follow-up was needed to recheck the video electroencephalogram to exclude epilepsy. In future follow-up, attention should be paid to the electroencephalogram results, to ensure early detection, early diagnosis, and early treatment.

At present, there are no long-term follow-up reports of patients with type 2 Noonan syndrome with hair loosening. Through regular follow-up, we found that active comprehensive rehabilitation therapy could improve the patient's motor, language, social, and cognitive skills. Comprehensive rehabilitation training including physical therapy, occupational therapy, speech therapy, sensory integration training, and special education can improve the level of intellectual function, social life ability, and self-care ability of children, and reduce the degree of treatment barriers and limited participation. Although no serious complications have occurred in our patient up to the time of writing, it is still necessary to follow-up the organ function of each system and carry out Multidisciplinary teamwork of the children are normal, and the risk of rebearing children with NSLH2 is small. However, we still need to be alert regarding reproductive chimerism. Genotypes and phenotypes caused by RASopathies are better understood now than before and hence, it is relatively easy for families for obtain a prenatal diagnosis. However, the new variation of prenatal diagnosis still has certain limitations, thus, clinicians should increase alertness, carefully check when prenatal diagnosis fetal face, once found, wide eyes, short nose forehead the special features such as, low set ears, should be on high alert, genetic tests in a timely manner.

In conclusion, we report a novel variant c.371A>G in exon 3 of the *PPP1CB* gene in a young pediatric patient with NSLH2. This study enriched the gene mutation spectrum of *PPP1CB*, and provided some reference for the diagnosis of this syndrome. To our knowledge, this is the first Chinese study to report this novel mutation in *PPP1CB* in NSLH2; therefore, further functional studies are needed to confirm the underlying pathogenic mechanism.

## Ethics statement

Written informed consent was obtained from the individual' legal guardian/next of kin, for the publication of any potentially identifiable images or data included in this article.

## Author contributions

XH, XM, JW, JM, and YL conceived this study, contributed to the study design, and attributed to project management. XH, XM, and JW wrote this manuscript and performed data collection. ZZ and CL generated the figures and tables. HH and JR contributed to guidance on English writing and performed data analysis. NZ carried out the literature search. XH and JW revised the manuscript. All authors have read and approved the content of the manuscript.

## Funding

This study was supported by the Kunming Medical University Joint Project-the Science and Technology Planning Project of the Yunnan Provincial Department of Science and Technology (Nos. 202101AY070001-215 and 202201AY070001-205), the Scientific Research Fund of Yunnan Provincial Education Department (No. 2022J0202), and Kunming Health Science and Technology Talent Training Project [No. 2022- SW (back-up)-005].

## Conflict of interest

The authors declare that the research was conducted in the absence of any commercial or financial relationships that could be construed as a potential conflict of interest.

## Publisher's note

All claims expressed in this article are solely those of the authors and do not necessarily represent those of their affiliated organizations, or those of the publisher, the editors and the reviewers. Any product that may be evaluated in this article, or claim that may be made by its manufacturer, is not guaranteed or endorsed by the publisher.
